# Effects of glucose metabolism pathways on nuclear and cytoplasmic maturation of pig oocytes

**DOI:** 10.1038/s41598-020-59709-6

**Published:** 2020-02-17

**Authors:** Jing Wen, Guo-Liang Wang, Hong-Jie Yuan, Jie Zhang, Hong-Li Xie, Shuai Gong, Xiao Han, Jing-He Tan

**Affiliations:** 1 Shandong Provincial Key Laboratory of Animal Biotechnology and Disease Control and Prevention, College of Animal Science and Veterinary Medicine, Shandong Agricultural University, Tai’an City, 271018 P.R. China; 2 College of Life Science, Northeast Agricultural University, Harbin, 150030 P.R. China

**Keywords:** Biochemistry, Cell biology, Developmental biology

## Abstract

The developmental competence of IVM porcine oocytes is still low compared with that in their *in vivo* counterparts. Although many studies reported effects of glucose metabolism (GM) on oocyte nuclear maturation, few reported on cytoplasmic maturation. Previous studies could not differentiate whether GM of cumulus cells (CCs) or that of cumulus-denuded oocytes (DOs) supported oocyte maturation. Furthermore, species differences in oocyte GM are largely unknown. Our aim was to address these issues by using enzyme activity inhibitors, RNAi gene silencing and special media that could support nuclear but not cytoplasmic maturation when GM was inhibited. The results showed that GM in CCs promoted pig oocyte maturation by releasing metabolites from both pentose phosphate pathway and glycolysis. Both pyruvate and lactate were transferred into pig DOs by monocarboxylate transporter and pyruvate was further delivered into mitochondria by mitochondrial pyruvate carrier in both pig DOs and CCs. In both pig DOs and CCs, pyruvate and lactate were utilized through mitochondrial electron transport and LDH-catalyzed oxidation to pyruvate, respectively. Pig and mouse DOs differed in their CC dependency for glucose, pyruvate and lactate utilization. While mouse DOs could not, pig DOs could use the lactate-derived pyruvate.

## Introduction

*In vitro* maturation (IVM) can provide large numbers of competent oocytes for embryo technology studies as well as for livestock production and human clinical practice^1^. It is anticipated that genetically engineered pigs will increasingly be used in biomedical research, because the pigs share many similarities with humans in terms of physiology, metabolism, genome organization, pathology and aging^2,3^. However, despite great efforts to make improvements, the developmental competence of IVM porcine oocytes is still low compared with that of their counterparts *in vivo* and in bovine and mouse^4–6^. Further observations indicated that the impaired developmental capacity of IVM oocytes were due mainly to an insufficient cytoplasmic maturation^7^.

The process of oocyte maturation includes both nuclear and cytoplasmic aspects^8,9^. Studies have demonstrated that progression through all the dynamic processes during oocyte maturation requires a large quantity of energy from metabolism of carbohydrates, amino acids and lipids^10,11^. Both meiosis resumption^12,13^ and the progression of meiosis to metaphase II stage^14,15^ are associated with increased glucose metabolism (GM) through one or more pathways. However, although there have been many reports on the effect of GM on oocyte nuclear maturation^16,17^, studies on GM effect on cytoplasmic maturation are limited. In the few studies reporting the GM effect on cytoplasmic maturation, the effect was analyzed together with its effect on nuclear maturation^18–20^. Furthermore, in all the previous studies addressing roles of GM and its metabolites on oocyte maturation, intact cumulus-oocyte complexes (COCs) were treated with enzyme inhibitors or stimulators. Because inhibitors/stimulators may have non-specificity and/or toxicity, and culture of COCs cannot differentiate whether GM of cumulus cells (CCs) or that of the cumulus-denuded oocytes (DOs) supports oocyte maturation, the results from previous studies remain to be verified by silencing specific genes in either CCs or DOs.

Pig oocytes differ from those of other species in containing a large quantity of endogenous lipid. For example, whereas a mouse oocyte typically contains only 4 ng of lipid^21^, an immature pig oocyte contains 156 ng lipid^22^. In mouse oocytes, inhibition and stimulation of fatty acid β-oxidation increased and decreased glucose consumption, respectively, suggesting that fatty acid metabolism and GM are correlated in the oocyte^23^. Furthermore, stimulation of lipid metabolism by l-carnitine could partially compensate for deficiencies in carbohydrate provision^24^. Thus, oocyte GM in pigs might be different from that in other species, which necessitates a special research. The pathways by which pyruvate and lactate are utilized during maturation of pig oocytes have not been reported. Furthermore, species differences in oocyte GM are largely unknown.

In this study, effects of GM on cytoplasmic maturation of pig oocytes were studied using special maturation media that could support nuclear maturation but could not support cytoplasmic maturation when GM was inhibited; whether GM in pig CCs or DOs supported oocyte maturation was differentiated by RNAi gene silencing; and the capacity to utilize glucose, pyruvate and lactate was compared between pig and mouse DOs. The results suggested that GM in CCs is essential for oocyte cytoplasmic maturation and that there are significant species differences in energy substrate metabolism between pig and mouse DOs.

## Results

### Formulation of the maturation medium for evaluating cytoplasmic maturation of pig COCs

**Figure 1:**
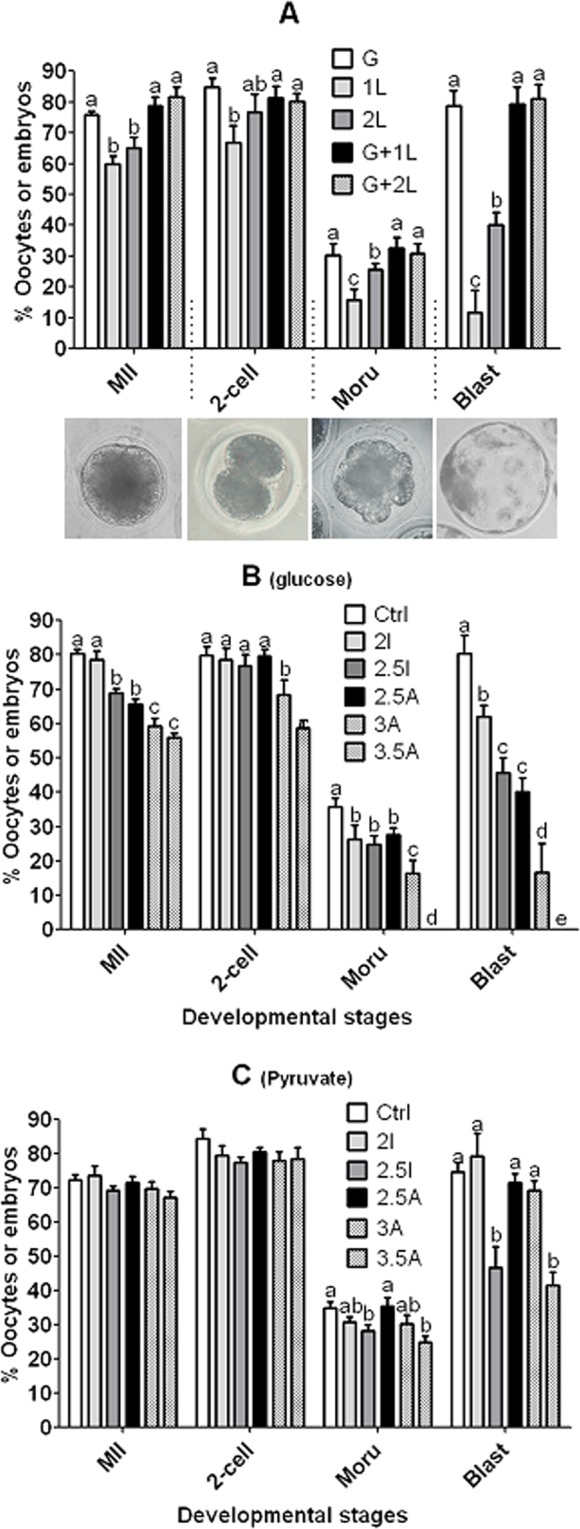
Effects of inhibiting glycolysis or PPP with iodoacetate or 6-AN, respectively, on nuclear maturation and developmental capacity of pig oocytes. Pig COCs were matured for 48 h in different media. In graph (**A**), oocytes were matured in the NCSU-23 medium supplemented with 5.6 mM glucose alone (G), with 1 or 2 mM lactate alone (1 L or 2 L), or with 5.6 mM glucose plus 1 or 2 mM lactate (G + 1 L or G + 2 L). In graph (**B**), oocytes were matured in control (Ctrl) medium (NCSU-23 supplemented with 5.6 mM glucose and 1 mM lactate) alone, or with 2 or 2.5 µM iodoacetate (2I or 2.5I), or with 2.5, 3 or 3.5 µM 6-AN (2.5A, 3A or 3.5A). In graph (**C**), oocytes were matured in control (Ctrl) medium (NCSU-23 containing 15 mM pyruvate) alone, or with 2 or 2.5 µM iodoacetate (2I or 2.5I), or with 2.5, 3 or 3.5 µM 6-AN (2.5A, 3A or 3.5A). At the end of the maturation culture, COCs were freed of CCs and those with a first polar body were activated for embryo culture. Each treatment was repeated 5 to 7 times with each replicate containing about 20 oocytes. The percentages of metaphase II (MII) oocytes, 2-cell embryos, morulae (Moru) and blastocysts (Blast) were calculated from oocytes cultured, MII oocytes, 2-cell embryos and morulae, respectively. a–e: Values without a common letter above bars differ significantly (P < 0.05) within developmental stages.

To establish a maturation medium that could sustain nuclear maturation without glucose but could not support blastocyst formation in glucose absence, the NCSU-23 medium that does not contain any energy substrate was chosen as the base medium. Then, pig COCs were matured for 48 h in NCSU-23 supplemented with glucose or lactate alone or in combination. At the end of the maturation culture, the COCs were freed of CCs and those oocytes showing a first polar body were considered mature (MII) and selected for parthenogenetic activation and embryo culture. When cultured with lactate alone, although both 1 and 2 mM lactate supported a similar maturation rate of around 60%, while 1 mM lactate produced only 10% blastocysts, 2 mM lactate generated 40% of blastocysts (Fig. 1A). When cultured with both glucose and 1 or 2 mM lactate, blastocyst rates increased to the same level as in oocytes matured with glucose alone, suggesting that lactate did not interfere with metabolism of glucose when used at these concentrations. Thus, the NCSU-23 medium containing 1 mM lactate was chosen as a maturation medium that could support acceptable nuclear maturation of pig COCs but sustained only a limited blastocyst formation without glucose.

### Effects of blocking glycolysis or PPP on cytoplasmic maturation of pig COCs

Pig COCs were matured for 48 h in a control medium (NCSU-23 supplemented with 5.6 mM glucose and 1 mM lactate) alone, or with different concentrations of glycolysis inhibitor, iodoacetate or PPP inhibitor, 6-AN. At the end of the maturation culture, the COCs were freed of CCs and those with a first polar body were activated for embryo development. After maturation of oocytes with different concentrations of iodoacetate or 6-AN, both rates of morulae and blastocysts decreased significantly with increasing drug concentrations, compared to those in oocytes matured in control medium without inhibitors (Fig. 1B). To rule out the possibility that iodoacetate and 6-AN impaired oocyte competence by their cellular toxicity, COCs were matured for 48 h in a control medium (NCSU-23 containing 15 mM pyruvate) alone or with different concentrations of iodoacetate or 6-AN. Among the concentrations observed, only 2.5 µM iodoacetate and 3.5 µM 6-AN affected rates of morulae and blastocysts in the presence of pyruvate (Fig. 1C). Furthermore, when morula and blastocyst rates were compared between the non-toxic concentrations of 2 µM iodoacetate and 3 µM 6-AN in the presence of glucose, it was found that morula (26% vs. 16%) and blastocyst rates (62% vs. 17%) were significantly higher in iodoacetate-treated than in the 6-AN-treated oocytes (Fig. 1B). Taken together, the results suggested that both PPP and glycolysis of GM were essential for cytoplasmic maturation of pig oocytes and that PPP was more important than glycolysis in this regard.

### The metabolic pathways for pyruvate and lactate to promote cytoplasmic maturation of pig COCs

**Figure 2:**
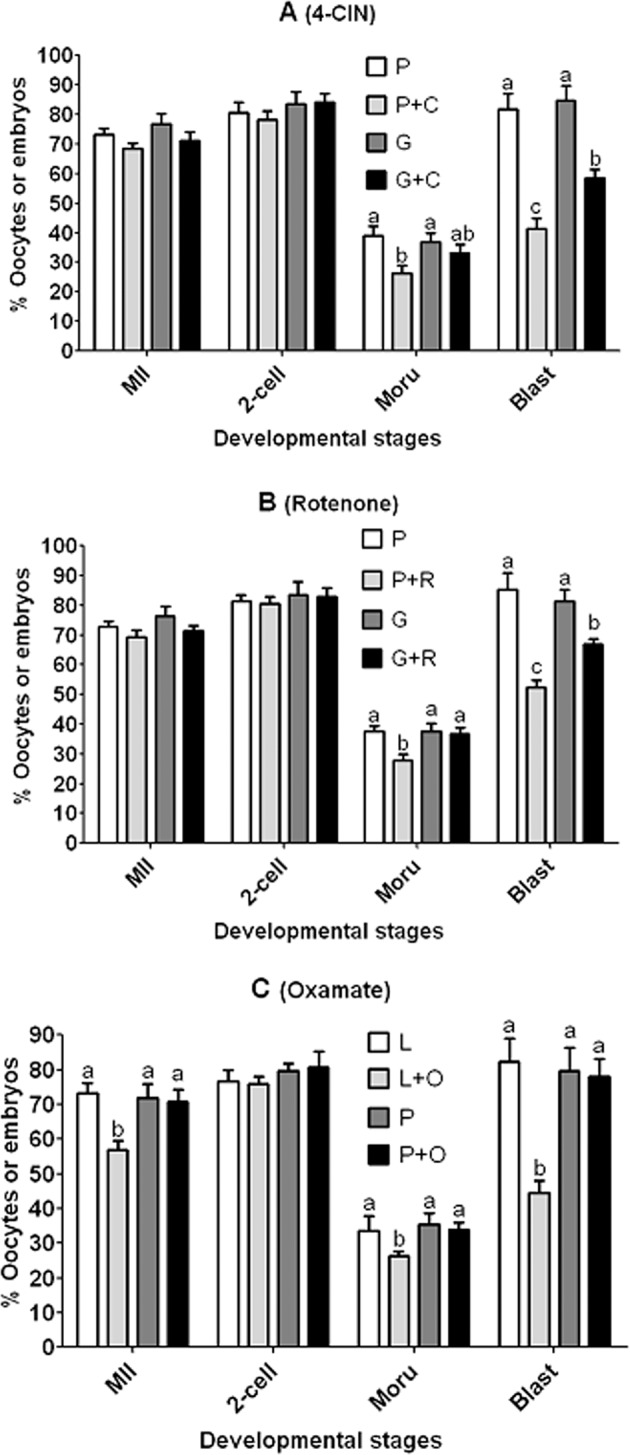
Nuclear maturation and developmental potential of pig COCs after inhibiting monocarboxylate transporter (MCT), mitochondrial respiratory chain, or lactate dehydrogenase with α-cyano-4-hydroxy cinnamate (4-CIN), rotenone, or sodium oxamate, respectively. In panel A, pig COCs were cultured for 48 h in NCSU-23 medium containing 15 mM pyruvate (P) or 5.6 mM glucose (G) alone, or with 20 µM of 4-CIN (P or G + C). In panel B, pig COCs were matured for 48 h in NCSU-23 containing P or G alone or with 0.05 µM rotenone (P or G + R). In panel C, pig COCs were matured for 48 h in NCSU-23 containing 3 mM lactate (L) or P alone or with 50 mM oxamate (L or P + O). At the end of the maturation culture, the COCs were freed of CCs and those with a first polar body were selected for activation and embryo culture. Each treatment was repeated 5 to 7 times with each replicate containing about 20 oocytes. The percentages of metaphase II (MII) oocytes, 2-cell embryos, morulae (Moru) and blastocysts (Blast) were calculated from cultured oocytes, MII oocytes, 2-cell embryos and morulae, respectively. a–d: Values without a common letter above bars differ significantly (P < 0.05) within developmental stages.

Pig COCs were cultured for 48 h in NCSU-23 medium containing pyruvate, glucose or lactate alone or together with monocarboxylate transporter (MCT) inhibitor, 4-CIN, mitochondrial respiratory chain inhibitor, rotenone or lactate dehydrogenase inhibitor, sodium oxamate before examination for nuclear maturation and embryo development. Blastocyst rates were significantly lower when COCs were matured with pyruvate plus 4-CIN or rotenone than with pyruvate alone (Fig. 2A,B). Although blastocyst rates in COCs matured with glucose plus 4-CIN or rotenone were also significantly lower than those matured with glucose alone, they were significantly higher than those matured with pyruvate plus 4-CIN or rotenone. Furthermore, although morula rates were significantly lower with pyruvate plus 4-CIN or rotenone than with pyruvate alone, they did not differ significantly between glucose alone and glucose with 4-CIN or rotenone. The presence of sodium oxamate significantly decreased rates of morulae and blastocysts when the maturation medium contained lactate but it did not affect morula and blastocyst rates when the maturation medium contained pyruvate (Fig. 2C). Furthermore, pyruvate, glucose and lactate produced similar rates of morulae and blastocysts when used alone. Taken together, the results suggested that (i) pyruvate was transported by MCT and metabolized through the mitochondrial respiratory chain to promote cytoplasmic maturation of pig oocytes; (ii) glucose supported cytoplasmic maturation of pig oocytes not only by producing pyruvate but also through other pathways; (iii) lactate metabolism in COCs supported cytoplasmic maturation via LDH-catalyzed oxidation to pyruvate; (iv) 4-CIN, rotenone and sodium oxamate were non-toxic when used at the current concentrations; and (v) pig COCs had an equal ability to utilize glucose, pyruvate or lactate for cytoplasmic maturation.

### Establishment of the maturation system for evaluating the effects of GM in CCs on nuclear and cytoplasmic maturation of pig DOs

**Figure 3:**
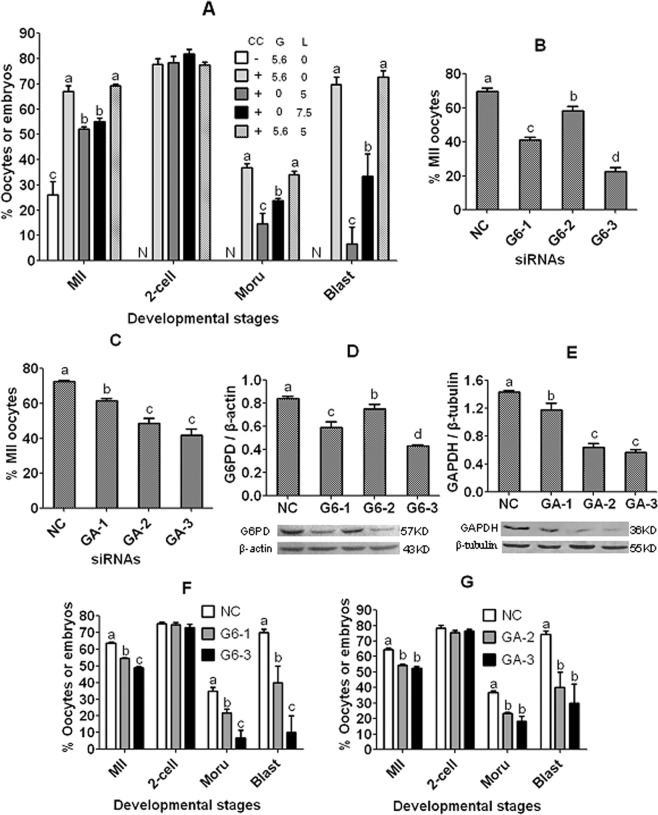
Effects of silencing G6PD or GAPDH mRNAs in CCM on maturation and developmental potential of co-cultured DOs. Graph (**A**) shows maturation and embryo development after pig DOs were cultured for 48 h with (+) or without (−) CCM (CC) in the NCSU-23 medium containing different concentrations (mM) of glucose (G) and/or lactate (L). Graphs (**B,C**) show percentages of mature (MII) DOs/co-cultured DOs, graphs (**D,E**) show protein levels of G6PD and GAPDH in CCM, and graphs (**F,G**) show developmental potential of cocultured DOs, respectively, after silencing G6PD or GAPDH mRNAs in CCM. The genes were silenced by transfection of CCM with negative control (NC) siRNA, G6PD siRNA-1 (G6-1), -2, or -3, or GAPDH siRNA-1 (GA-1), -2, or -3. In graphs (**A,B,C,F,G**), percentages of matured (MII) oocytes, 2-cell embryos, morulae and blastocysts were calculated from oocytes cultured, MII oocytes, 2-cell embryos and morulae, respectively. Each treatment was repeated 3–5 times with each replicate containing about 20 DOs. In graphs (**D,E**), the ratio of G6PD/β-actin or GAPDH/β-tubulin was used to indicate the level of protein expression in CCM. In graphs (**B,C**), DOs were cocultured for 48 h on gene knockdown CCM in the NCSU-23 medium containing 5.6 mM glucose alone, whereas in graphs (**F,G**), DOs were cocultured on gene knockdown CCM in NCSU-23 with both 5.6 mM glucose and 5 mM lactate. a–d: Values with a different letter above bars differ significantly (p < 0.05) within developmental stages. Embryo development was not observed (N) in DOs matured without CCM due to the limited number of mature oocytes available (Graph **A**). For original images of gels/blots from Western blotting, please refer to Supplemental Information as provided.

To differentiate whether the GM of CCs or that of oocyte itself supports oocyte nuclear and cytoplasmic maturation of pig oocytes, a coculture maturation system that allows evaluation of the effect of GM in CCs on nuclear and cytoplasmic maturation of DOs was first established. Pig DOs were cocultured with CC monolayer (CCM) in the presence of glucose or lactate alone or in combination for 48 h before examination for nuclear and cytoplasmic maturation. While 5.6 mM glucose supported nuclear maturation in only 26% of DOs when cultured without CCM, the nuclear maturation rate increased to 67% when cultured with both glucose and CCM (Fig. 3A). This indicated that pig DOs had a limited capacity to use glucose and that CCs supported nuclear maturation of cocultured DOs by metabolizing glucose, suggesting that the CCM + 5.6 mM glucose system can be used to evaluate nuclear maturation of cocultured DOs. Although both 5 and 7.5 mM lactate supported a similar rate of nuclear maturation, 5 mM lactate produced significantly lower rates of morulae and blastocysts than 7.5 mM lactate did. Furthermore, when 5 mM lactate was added together with 5.6 mM glucose, rates of nuclear maturation, morulae and blastocysts were all similar to those obtained with addition of glucose alone. Thus, the CCM + 5.6 mM glucose + 5 mM lactate protocol can be used to examine cytoplasmic maturation of cocultured DOs.

### Effects of RNAi-knockdown of G6PD or GAPDH expression in CCs on nuclear and cytoplasmic maturation of cocultured pig DOs

Three siRNA sequences were used to silence the G6PD gene and three were used to silence the GAPDH gene. On reaching about 80% of confluence at 24 h of the CCs culture, the CCM was transfected with negative control (NC), G6PD or GAPDH siRNAs. After 48 h of transfection, the CCM was used either for coculture of DOs or for western analysis of gene silencing efficiency. To observe nuclear maturation, pig DOs were cultured for 48 h on the transfected CCM in NCSU-23 medium containing 5.6 mM glucose. Transfection of CCM with G6PD siRNA-1 or -3, or GAPDH siRNA-2 or -3 significantly decreased nuclear maturation of the cocultured DOs, whereas transfection with G6PD siRNA-2 or GAPDH siRNA-1 showed a mild effect (Fig. 3B,C). Western analysis further confirmed the high gene silencing efficiency for G6PD siRNA-1 and -3, and for GAPDH siRNA-2 and -3, compared to the other siRNA sequences tested (Fig. 3D,E). It should be noted that the lowest nuclear maturation rate achieved with GAPDH siRNA-3 (41.9 ± 3.5%) was still higher significantly than that observed with G6PD siRNA-3 (22.7 ± 2.2%).

To observe cytoplasmic maturation, pig DOs were cultured for 48 h on the transfected CCM in NCSU-23 medium containing 5.6 mM glucose and 5 mM lactate. At the end of the maturation culture, matured oocytes were activated for embryo development. The results showed that rates of MII oocytes, morulae and blastocysts were all significantly lower in oocytes matured on CCM transfected with G6PD siRNA-3 than with G6PD siRNA-1 than with negative control siRNA (Fig. 3F). Similarly, rates of MII oocytes, morulae and blastocysts were all significantly lower in oocytes matured on CCM transfected with GAPDH siRNA-2 or -3 than with negative control siRNA (Fig. 3G). It should be noted that the lowest morula (18.4 ± 3.0%) and blastocyst rates (30.0 ± 12.2%) achieved with GAPDH siRNA-3 were still significantly higher than those obtained with G6PD siRNA-3 (6.9 ± 4.5% of morulae and 10.0 ± 10.0% of blastocysts). Taken together, the results suggested that both GM through PPP and glycolysis in CCs was important for nuclear and cytoplasmic maturation of pig oocytes and that PPP played a more important role than glycolysis did in this regard.

### Effects of down regulating enzymes for pyruvate or lactate metabolism in CCM or DOs on ***in vitro*** maturation of pig DOs

**Figure 4:**
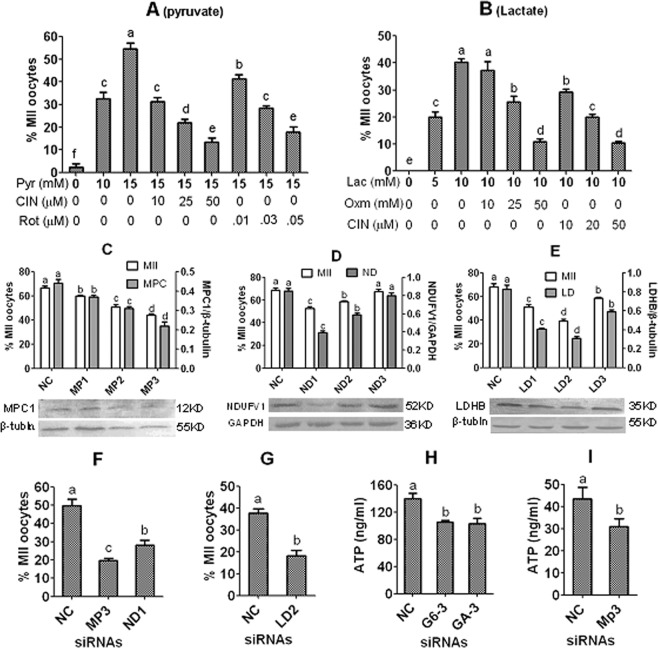
Effects of down regulating related genes in DOs or CCM on *in vitro* maturation of pig oocytes. Graph (**A**) shows percentages of MII oocytes after DOs were cultured for 48 h in the NCSU-23 medium containing 0, 10 or 15 mM pyruvate (Pyr) with 10, 25 or 50 µM 4-CIN (CIN) or with 0.01, 0.03 or 0.05 µM rotenone (Rot). Graph B shows percentages of MII oocytes after DOs were cultured for 48 h in the NCSU-23 medium containing 0, 5 or 10 mM lactate (Lac) with 10, 25 or 50 mM sodium oxamate (Oxm) or with 10, 20 or 50 µM 4-CIN. Graphs (**C–E**) show MII percentages of cocultured DOs (Left Y axis) and levels of MPC1, NDUFV1 and LDHB (Right Y axis) after transfection of CCM with negative control (NC) siRNA, MPC1 siRNA (MP1, 2 or 3), NDUFV1 siRNA (ND1, 2 or 3), or LDHB siRNA (LD1, 2 or 3), respectively. Graphs (**F,G**) show MII percentages of DOs after microinjection with NC, MP3, ND1 or LD2 siRNAs. While DOs and the CCM transfected/injected with MPC1 or NDUFV1 siRNAs were cultured in NCSU-23 containing 15 mM pyruvate, DOs and the CCM transfected/injected with LDHB siRNA were cultured in NCSU-23 containing 10 mM lactate. In oocyte maturation experiments, each treatment was repeated 4 times with each replicate containing about 20 oocytes. Graphs (**H,I**) show ATP concentrations (ng/ml) in medium conditioned with CCM in NCSU-23 medium containing 5.6 mM glucose and 15 mM pyruvate, respectively, after transfection with G6PD siRNA-3 (G6-3), GAPDH siRNA-3 (GA-3) or MPC1 siRNA-3 (MP3). Each treatment was repeated 3 times with each replicate containing medium recovered from one culture well on different experimental days. a–f: Values with a different letter above bars differ significantly (P < 0.05).

Expression or activities of MCT, mitochondrial pyruvate carrier 1 (MPC1), mitochondrial electron transport chain (ETC), NADH dehydrogenase (ubiquintone) flavoprotein 1 (NDUFV1) and lactate dehydrogenase (LDHB) were down regulated in DOs or CCM to observe the effects of pyruvate and lactate metabolism on maturation of pig DOs. Firstly, the activities of MCT, ETC and LDH were down regulated with 4-CIN, rotenone and sodium oxamate, respectively. Pig DOs were cultured for 48 h in the NCSU-23 medium containing different concentrations of pyruvate or lactate with various concentrations of the inhibitors before examination for nuclear maturation. The results showed that the optimal concentration of pyruvate was 15 mM, which supported nuclear maturation in about 55% of the DOs (Fig. 4A). The nuclear maturation rates of DOs decreased significantly with increasing concentrations of MCT inhibitor, 4-CIN or ETC inhibitor, rotenone in the presence of 15 mM pyruvate. The optimal concentration of lactate was 10 mM, which sustained a nuclear maturation rate of about 40% in pig DOs (Fig. 4B). The nuclear maturation rates of DOs declined significantly with increasing concentrations of the LDH inhibitor, sodium oxamate or MCT inhibitor, 4-CIN in the presence of 10 mM lactate.

Secondly, expression of the MPC1, NDUFV1 and LDHB genes in CCM were silenced by transfection with respective siRNA sequences, and then, DOs were cocultured on the CCM transfected with MPC1 or NDUFV1 siRNAs or with LDHB siRNA in NCSU-23 containing 15 mM pyruvate or 10 mM lactate, respectively, to observe nuclear maturation. Transfection of CCM with either MPC1, NDUFV1 or LDHB siRNAs impaired maturation of cocultured DOs and the impairment was most significant with MPC1 siRNA3 (MP3), NDUFV1 siRNA1 (ND1) and LDHB siRNA2 (LD2) (Fig. 4C–E). Our western blotting further confirmed the high gene silencing efficiency for MP3, ND1 and LD2 compared with other siRNAs tested.

Thirdly, expression of the MPC1, NDUFV1 and LDHB genes in DOs were silenced by microinjection of MP3, ND1 and LD2, respectively. While DOs injected with MP3 or ND1 were cultured for 48 h in NCSU-23 containing 15 mM pyruvate, DOs injected with LD2 were cultured in NCSU-23 containing 10 mM lactate before examination for nuclear maturation. The results showed that maturation rates were significantly lower after injection of either MP3, ND1 or LD2 siRNA than injection of NC siRNA (Fig. 4F,G). Taken together, the results suggested that pig DOs could metabolize pyruvate and lactate to support a certain degree of nuclear maturation; both pyruvate and lactate were transported into the DOs through MCT; pyruvate was transferred into mitochondria through MPC in both DOs and CCs; and while pyruvate was metabolized through ETC, lactate was utilized via the LDH-catalyzed oxidation to pyruvate to support oocyte maturation in both pig DOs and CCs.

### Pig CCs released ATP into the culture medium

The above results from gene silencing experiments suggested that CCM promoted pig oocyte maturation by releasing metabolites into culture medium. To confirm this release of metabolites, we measured the ATP contents in medium conditioned by CCM after silencing related genes. Silencing G6PD or GAPDH gene in CCM significantly decreased the ATP level in medium conditioned in the presence of glucose (Fig. 4H), and knocking down MPC1 gene significantly reduced ATP release from CCM cultured with pyruvate (Fig. 4I).

### Pig and mouse DOs differ in their dependencies on CCs for pyruvate, lactate and glucose utilization

When percentages of MII oocytes were compared between pig or mouse COCs and DOs after maturation culture in basic medium containing optimal concentrations of pyruvate, lactate or glucose, it was found that although maximum maturation was achieved in COCs of both species, maturation rates of DOs differed significantly between the two species after culture with either pyruvate, lactate or glucose (Table 1). This suggested that pig and mouse DOs might differ in their dependencies on CCs for glucose, pyruvate and lactate utilization to support maturation. Experiments were thus conducted to verify this species difference.

**Table 1:** Percentages of MII oocytes and blastocysts after pig or mouse COCs or DOs were matured in basic maturation medium^a^ containing optimal concentrations of pyruvate, lactate or glucose^b^.

Oocytes	Energy	Pyruvate		Lactate		Glucose	
	Species	Pig	Mouse	Pig	Mouse	Pig	Mouse
	Concent. (mM)^c^	15	2	3/10	5	5.6	5.6
COCs	**% MII oocytes**	**72**	**100**	**72**	**100**	**75**	**98**
	% Blastocyst	80	30	80	21^d^	78	34
DOs	**% MII oocytes**	**55**	**100**	**40**	**0**	**25**	**0**
	% Blastocyst	NE	8.5	NE	NE	NE	NE

^a^Simplified α-MEM for mouse oocytes and NCSU-23 medium for pig oocytes. ^b^Data for pig oocytes were obtained in this study, whereas those for mouse oocytes were reported by Xie *et al*.^26^. In experiments with pig oocytes, each treatment was repeated 4-5 times with each replicate including around 20 oocytes. ^c^Concentrations of pyruvate, lactate and glucose used for culture of pig or mouse COCs or DOs. The concentration of lactate for culture of COCs and DOs were 3 and 10 mM, respectively. ^d^Significantly (P < 0.05) different from the maximum rates of blastocysts in mouse COCs obtained with pyruvate (30%) or glucose (34%). “NE” Not examined.

**Figure 5:**
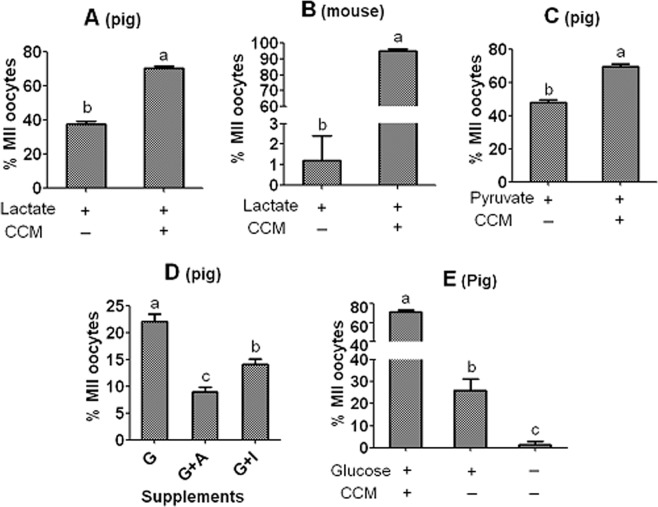
Effects of coculture on CCM with lactate, pyruvate or glucose on nuclear maturation of pig and mouse DOs. Graphs (**A** or **C** and **B**) show percentages of MII oocytes after maturation of pig DOs for 48 h in NCSU-23 medium containing 10 mM lactate and mouse DOs for 15 h in α-MEM medium containing 2 mM lactate, respectively, with (+) or without (−) CCM. Graph D shows percentages of MII oocytes after pig DOs were matured for 48 h in NCSU-23 containing 5.6 mM glucose (G) without or with (+) 3 µM 6-AN (**A**) or 2 µM iodoacetate (I). Graph E shows percentages of MII oocytes after pig DOs were matured for 48 h in NCSU-23 with (+) or without (−) 5.6 mM glucose and/or CCM. Each treatment was repeated 4 times with each replicate containing about 20 oocytes. a–c: Values with a different letter above bars differ significantly (P < 0.05).

Pig and mouse DOs were cocultured with CCM in NCSU-23 or alpha-MEM medium containing optimal concentrations of pyruvate, lactate or glucose for 48 h or 15 h, respectively, before examination for nuclear maturation. The MII percentage was 38% and 1.2%, respectively when pig and mouse DOs were cultured with lactate without CCM, but it increased to a maximum of 71% and 95%, respectively, when cultured with CCM (Fig. 5A,B). When cultured with pyruvate without CCM, while 100% of mouse DOs matured (Table 1), only 48% of pig DOs attained nuclear maturation (Fig. 5C). When cultured with both pyruvate and CCM, however, a maximum nuclear maturation of 70% was observed in pig DOs. While 25% pig DOs developed to MII stage (Fig. 3A), none of the mouse DOs matured^25^ when cultured with glucose without CCM. When cultured with both glucose and CCM, however, the MII percentage increased to a maximum of about 70% and 95% in pig and mouse oocytes, respectively. Thus, pig DOs are more capable of metabolizing glucose and lactate but less capable of utilizing pyruvate to support maturation than mouse DOs are. In other words, compared to mouse DOs, pig DOs are less dependent on CCs in metabolizing glucose and lactate but more dependent on CCs in utilizing pyruvate to support maturation. Furthermore, when pig DOs were matured with glucose in the presence of either 6-AN or iodoacetate, the MII percentage decreased significantly compared to that when cultured with glucose alone (Fig. 5D), suggesting that glucose was utilized by pig DOs through PPP and glycolysis to support maturation. To study whether pig DOs could take in glucose directly from medium, they were cultured for 48 h in NCSU-23 with or without 5.6 mM glucose before examination for nuclear maturation. While 26% DOs matured in the presence of glucose, the maturation rate was only 1.4% without glucose (Fig. 5E), confirming that pig DOs could absorb glucose directly from medium. Taken together, the results confirmed that there is a significant species difference in oocytes’ dependencies on CCs for metabolism of energy substances to support their maturation.

### Pig DOs could use the lactate-derived pyruvate contrary to mouse DOs

**Figure 6:**
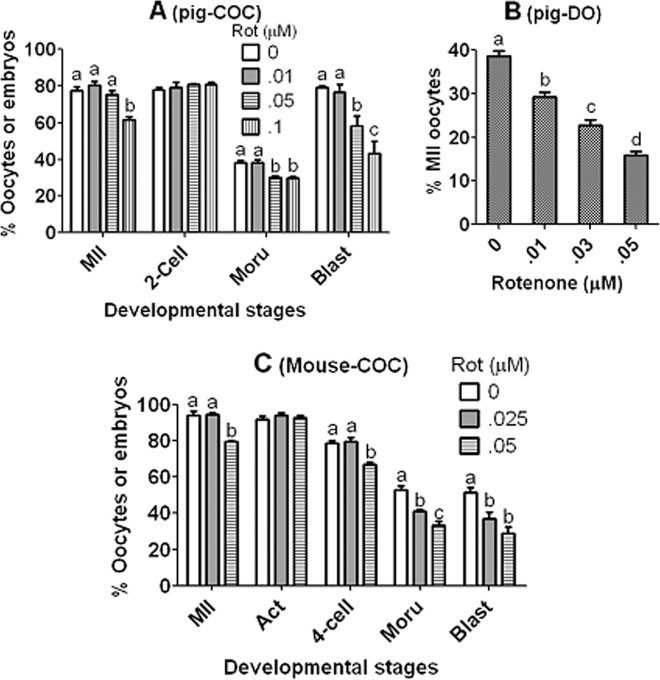
Utilization of lactate-derived pyruvate during maturation of pig or mouse COCs or DOs. Pig COCs (graph **A**) and DOs (graph **B**) were matured for 48 h in NCSU-23 medium containing 10 mM lactate and various concentrations (µM) of ETC inhibitor, rotenone. Mouse COCs (graph **C**) were matured for 15 h in alpha-MEM medium containing 2 mM lactate and different concentrations (µM) of rotenone. At the end of maturation, oocytes were examined for maturation and activated for embryo development. Each treatment was repeated 5 times with each replicate containing about 20 oocytes. a–d: Values with a different letter above bars differ significantly (P < 0.05).

Our previous study demonstrated that blastocyst rates were significantly lower after maturation of mouse COCs with lactate than with pyruvate or glucose and that while 100% mouse DOs matured with pyruvate, none matured with lactate^26^ (Table 1). Our explanation for this poor lactate utilization of mouse oocytes was that whereas the culture medium- and glycolysis-derived pyruvate could enter the tricarboxylic acid (TCA) cycle, the lactate-derived pyruvate was poorly metabolized by mitochondria. However, the present results showed that pig COCs had an equal ability to use glucose, pyruvate and lactate for nuclear and cytoplasmic maturation and that 40% pig DOs could mature in the presence of lactate alone. This suggests that different from mouse oocytes, the pig oocytes could use the lactate-derived pyruvate for maturation. Experiments were thus performed to test this hypothesis. Pig COCs or DOs were matured for 48 h in NCSU-23 medium containing 10 mM lactate and different concentrations of ETC inhibitor, rotenone before examination for maturation and activation for embryo development. Rates of morulae and blastocysts in COCs and maturation rate in DOs decreased significantly with increasing concentrations of rotenone (Fig. 6A,B). However, rotenone also affected maturation and developmental potential of mouse COCs in the presence of lactate (Fig. 6C). Rotenone was nontoxic to pig COCs when used at 0.05 µM (Fig. 2B) and to mouse COCs when used at 0.025 µM^26^. Thus, the results confirmed that although both pig and mouse CCs could use the lactate-derived pyruvate to support oocyte maturation, mouse DOs could not utilize the lactate-derived pyruvate while pig DOs could.

## Discussion

Both our present results by culturing COCs with enzyme activity inhibitors and by RNAi gene silencing in CCs suggested that the GM in CCs promoted pig oocyte maturation by releasing metabolites from PPP and glycolysis. Although both PPP and glycolysis in CCs were essential, PPP played a more important role than glycolysis did in promoting oocyte maturation. Our further observation indicated that silencing either G6PD, GAPDH or MPC1 gene in CCM significantly decreased the ATP level in conditioned medium, confirming that CCM promoted pig oocyte maturation by releasing metabolites into culture medium. Similar results were obtained in our previous studies using the mouse oocyte model. For example, by culturing COCs with enzyme inhibitors, Xie *et al*.^26^ observed that while glycolysis promoted cytoplasmic maturation mainly by supplying energy, PPP facilitated cytoplasmic maturation to a greater extent through both reducing oxidative stress and supplying energy via providing glycolysis with fructose-6-phosphate. Xie *et al*.^25^ showed that although silencing either G6PD or GAPDH gene in CCs significantly impaired maturation of cocultured mouse DOs, silencing G6PD decreased oocyte competence to a greater extent. While silencing either G6PD or GAPDH in CCs decreased ATP contents of cocultured DOs to a similar extent, silencing G6PD significantly increased oxidative stress as well as decreasing ATP contents. Furthermore, by analyzing metabolite contents and oxidative stress index in medium conditioned with gene-silenced CCs and by culturing DOs in the conditioned medium, they observed that CCs supported oocyte maturation by releasing soluble glucose metabolites such as pyruvate, ATP, glutathione and NADPH.

The current results indicated that oocyte maturation with glucose produced significantly more morulae and blastocysts than oocyte maturation with pyruvate did in the presence of either rotenone or 4-CIN. Similar results were obtained with mouse oocytes in our previous studies^26^. It is known that the pathways of GM upstream the pyruvate generation also produce beneficial molecules. For instance, PPP produces NADPH and phosphoribosyl pyrophosphate (PRPP), and glycolysis produces ATP prior to pyruvate production. Downs^27,28^ reported that PRPP is involved in purine production and the resumption of meiosis. Furthermore, Downs *et al*.^29^ and Krisher^30^ found that the addition of PRPP to culture medium promoted oocyte maturation.

The present results showed that both pyruvate and lactate were transferred into DOs by MCT, and the pyruvate was further transported into mitochondria by MPC1. Both our experiments by culturing COCs with enzyme inhibitors and by RNAi gene silencing in DOs and CCs demonstrated that within DOs and CCs as well, while pyruvate was utilized through mitochondrial electron transport, lactate was metabolized through LDH-catalyzed oxidation to pyruvate. As far as we know, this is the first report on the pathways by which pyruvate and lactate are utilized during maturation of pig oocytes. Our recent studies on mouse oocytes showed that inhibiting MCT with 4-CIN or suppressing ETC with rotenone in COCs, or knocking down MPC1 or NDUFV1 genes with RNAi in DOs significantly impaired pyruvate utilization and oocyte maturation^25,26^. However, how lactate is metabolized in DOs and how pyruvate and lactate are utilized in CCs to support oocyte maturation was not observed in previous studies. Although expression of MCT has been observed in human and mouse^31^, bovine^32^ and Xenopus oocytes^33^, and it is recently established that MPC is located in the inner mitochondrial membrane and plays an essential role in mitochondrial pyruvate uptake in somatic cells^34^, the function of MCT and MPC during oocyte maturation had not been systematically studied before our studies. Furthermore, studies on ETC in oocytes of different species are few^35–37^, and the role of NDUFV1, the first complex in ETC that accepts electrons from NADH and is critical for ATP generation, has not been observed in oocytes. In addition, although expression of different LDH isozymes were observed in oocytes and/or CCs of cattle^38^, human^39^, mouse^40^ and buffalo^41^, their effects on oocyte maturation had not been carefully analyzed, and particularly, the effects had not been differentiated between DOs and CCs using such a powerful technique as RNAi.

The current results demonstrated that while pig DOs could utilize the lactate-derived pyruvate to promote their maturation, mouse DOs could not. However, both pig and mouse CCs could use the lactate-derived pyruvate to support oocyte maturation. The results are in consistency with those reported by Xie *et al*.^26^ who observed that blastocyst rates were significantly lower after maturation of mouse COCs with lactate than with pyruvate or glucose and that while 100% mouse DOs matured with pyruvate, none matured with lactate. Dumollard *et al*.^42^ also reported that the lactate-derived pyruvate was minimally metabolized by mitochondria of mouse oocytes, as mitochondrial oxidation was never affected by lactate addition. They proposed that there are two discrete pools of pyruvate inside the oocyte: one from the bathing medium, which is rapidly metabolized by the mitochondria, while the other from the lactate is poorly used by the mitochondria. Such an intracellular compartmentation of pyruvate pools has also been described in somatic cells^43,44^. However, Cetica *et al*.^38^ found that LDH-1 was the main isozyme in bovine oocytes and that the pyruvate used during oocyte maturation could be partly produced from lactate when the NAD supply was adequate, although their study used TCM-199 that contained glucose as the basic medium. Thus, the species difference in DO metabolization of lactate-derived pyruvate must be taken into account when formulating oocyte maturation medium for different species.

When maturation rates were compared between pig or mouse COCs and DOs after maturation culture in basic medium containing optimal concentrations of pyruvate, lactate or glucose, we found that although maximum maturation was achieved in COCs of both species, maturation rates of DOs differed significantly between the two species after culture with either pyruvate, lactate or glucose (Table 1). In this study we showed that although a maximum nuclear maturation was achieved in both species when DOs were cocultured with both CCM and glucose, pyruvate or lactate, maturation rates of DOs varied significantly with species when cultured without CCM. When cultured without CCM, for example, the MII percentages were 38% and 1.2%, 48% and 100%, and 25% and 0% in pig and mouse DOs, respectively, when cultured in the presence of lactate, pyruvate or glucose, respectively. Thus, compared to mouse DOs, the pig DOs are less dependent on CCs in metabolizing glucose and lactate but more dependent on CCs in utilizing pyruvate to support maturation. Cetica *et al*.^38^ observed maturation rates of 76.5% and 47.7%, 78.1% and 59.4%, and 74.5% and 47.6% after maturation of bovine COCs and DOs, respectively, in TCM-199 that contained glucose alone, glucose plus pyruvate or lactate, respectively. Thus, bovine DOs also show different dependencies on CCs for metabolization of glucose, pyruvate and lactate. Furthermore, the present results showed that pig DOs could take up glucose directly from medium, a result different from that reported by Wang *et al*.^45^ who observed in the mouse that treating COCs with glucose transporter (GLUT) inhibitors led to simultaneous decreases in glucose uptake in CCs and their surrounded oocyte but did not affect DOs. This might suggest a species difference in the GLUT system between pig and mouse oocytes. Again, this species difference in DOs’ dependency on CCs must be considered when formulating oocyte (particularly DO) maturation media for different species.

In summary, GM effects on cytoplasmic maturation of pig oocytes were studied using special maturation media that could support nuclear but not cytoplasmic maturation without glucose; whether GM in pig CCs or DOs supported maturation was differentiated by RNAi gene silencing; and the capacity to utilize glucose, pyruvate and lactate was compared between pig and mouse DOs. The results showed that in pig oocytes, GM in CCs promoted oocyte maturation by releasing metabolites from both PPP and glycolysis; pyruvate was transferred into DOs/CCs by MCT and MPC, and was utilized through ETC; lactate was delivered into DOs through MCT and utilized by LDH-catalyzed oxidation to pyruvate. Compared to mouse DOs, pig DOs were less dependent on CCs in metabolizing glucose and lactate but more dependent on CCs in utilizing pyruvate. While mouse DOs could not, pig DOs could use the lactate-derived pyruvate. The data must be taken into account when formulating oocyte (particularly DO) maturation medium for different species including human beings.

## Methods

The present study was carried out in accordance with the relevant guidelines and regulations. The methods used for animal care and handling were approved by the Animal Care and Use Committee of the Shandong Agricultural University P. R. China (Permit number: SDAUA-2001-001). Unless otherwise specified, all chemicals and reagents were purchased from Sigma Chemical Co. (St. Louis, MO, USA).

### Oocyte recovery

Porcine ovaries were obtained at the Feicheng slaughterhouse of Yinbao Food Corporation Ltd., Tai-an city, China. The ovaries were transported at 30–35 °C to the laboratory within 3 h after collection in a thermos bottle containing sterile saline with 100 IU/ml penicillin and 0.05 mg/ml streptomycin. COCs were recovered by aspirating 3–6 mm follicles using a syringe. The medium used for oocyte collection and washing was Dulbecco’s phosphate-buffered saline (D-PBS, HyClone, Logan, UT, USA) supplemented with 0.88 mM CaCl_2_·2H_2_O, 0.49 mM MgCl_2_·6H_2_O, 0.1% polyvinyl alcohol, 0.03 mM phenol red, 50 IU/ml penicillin and 50 µg/ml streptomycin. Only COCs with a uniform ooplasm and a compact cumulus were chosen for further treatment. To prepare DOs, the selected COCs were digested with 0.25% trypsin for 3 min, and then were pipetted several times to remove CCs. Procedures for mouse handling, oocyte recovery and DOs preparation are exactly those reported previously by this laboratory^25^.

### Oocyte maturation *in vitro*

The basic maturation medium was NCSU-23, which contained 108.73 mM NaCl, 4.78 mM KCl, 1.7 mM CaCl_2_·2H_2_O, 25.07 mM NaHCO_3_, 1.19 mM MgSO_4_·6H_2_O, 1.19 mM KH_2_PO_4_, 1.0 mM L-Glutamine, 7.0 mM Taurine, 5.0 mM Hypotaurine, 4.0 g/l BSA, 0.05 IU/ml FSH, 0.05 IU/ml LH, 10 ng/ml EGF, 0.57 mM Cysteine, 50 µg/ml streptomycin, and 100 IU/ml penicillin, but without any energy substrate. Various concentrations of energy substrates and metabolism regulators were added to the basic maturation medium according to the experimental design. To make stock solutions, α-cyano-4-hydroxy cinnamate (4-CIN, 100 mM) and rotenone (1 mM) were dissolved in dimethyl sulfoxide, whereas iodoacetate (4 mM) and 6-AN (100 mM) was dissolved in water. All the stock solutions were stored in aliquots at −20 °C and diluted to designed concentrations with the maturation medium immediately before use. When sodium lactate, sodium pyruvate and/or sodium oxamate was included in the medium, the osmotic pressure was adjusted accordingly by reducing the amount of sodium chloride. The maturation medium was placed in culture wells (150 µl per well) and pre-equilibrated at 38.5 °C in an atmosphere of 5% CO_2_ in humidified air for a minimum of 3 h before incubation of oocytes. After being washed three times in D-PBS and once in the maturation medium, COCs or DOs were placed in the wells (about 20 per well), covered with mineral oil, and cultured for 48 h at 38.5 °C under 5% CO_2_ in humidified air. The DOs were cultured either alone or on cumulus cell monolayer (CCM). After maturation culture, COCs were denuded of CCs by pipetting in D-PBS containing 0.1% hyaluronidase. The DOs obtained were examined under a microscope for maturation and those showing a first polar body were considered mature. Procedures and media used for *in vitro* maturation of mouse oocytes were exactly those reported previously by this laboratory^25^.

### Oocyte activation and embryo culture

Procedures for oocyte activation and embryo culture were those reported previously by Zhang *et al*.^46^. For activation, DOs were first treated with 5 µM ionomycin contained in D-PBS for 5 min. Then, the oocytes were washed three times with the PZM-3 medium and incubated for 5 h in PZM-3 medium containing 2 mM 6-DMAP. At the end of the incubation, 6-DMAP was removed by washing the oocytes in D-PBS and the activated oocytes were cultured for embryo development in PZM-3 at 38.5 °C in 5% CO_2_ in air. Cleavage rate was observed at 48 h of embryo culture. Morula and blastocyst rates were observed at 168 h of embryo culture under a fluorescence microscope after a 10-min staining with 10 µg/ml of Hoechst 33342 in D-PBS.

### Cumulus cell monolayer (CCM) preparation and small interfering RNA (siRNA) transfection

Procedures for CCM preparation and siRNA transfection were those reported previously^25^. Briefly, CCs that were released by pipetting COCs with a small-bore pipette were harvested and washed in a TCM-199 medium. After counting on a hemocytometer, the CCs were seeded in wells of a 96-well plate (2 × 10^5^ cells per well with 100 µl TCM-199 medium). The plate with CCs were then incubated at 38.5 °C in a humidified atmosphere of 5% CO_2_ in air. The TCM-199 medium (Gibco, Grand Island, New York, USA) were supplemented with 10% (v/v) fetal calf serum (Gibco), 1 µg/ml of 17β-estradiol, 0.22 mM sodium pyruvate, 0.05 IU/ml FSH, 0.05 IU/ml LH, 10 ng/ml epidermal growth factor (EGF), and ITS + 1 Liquid Media Supplement (I2521, Sigma, St. Louis, MO, USA). When the CCs grew to about 80% confluence around 24 h after seeding, the monolayers were used either for coculture of DOs, or for siRNA transfection. The siRNAs targeting mRNAs and the negative control siRNA were designed and synthesized by RiboBio (Guangzhou, China), and the sequences of their sense strands of targeting siRNAs are shown in Table 2. Transfection with 100 nM siRNAs was conducted using lipofectamine RNAiMAX reagent (Invitrogen/Life Technologies, Grand Island, NY) according to the manufacturer’s instructions. Thus, when the CCs grew to about 80% of confluence, the spent medium in the wells was replaced by 90 µl fresh TCM-199 medium and the cells were transfected by the forward transfection method. Briefly, 0.5 µl of a 20 µM solution of each siRNA were diluted in 4.5 µl of Opti-MEM medium (Invitrogen) and mixed with 0.3 µl of Lipofectamine RNAiMAX reagent (Invitrogen) diluted in 4.7 µl of Opti-MEM medium. After incubation for 5 min at room temperature, the transfection complex was added to the wells, and incubated for 48 h at 38.5 °C in a humidified 5% CO_2_ atmosphere.

**Table 2:** Sequences of the sense strands of siRNAs targeting different genes.

Genes	Targeting sequences
G6PD-siRNA-1 (G6-1)	5′-GCGTCATCCTCACCTTCAA-3′
G6PD-siRNA-2 (G6-2)	5′-CCTCATGGTGCTGAGGTTT-3′
G6PD-siRNA-3 (G6-3)	5′-GCTTTCCATCAGTCGGATA-3′
GAPDH-siRNA-1 (GA-1)	5′-GGTCTACATGTTCCAGTAT-3′
GAPDH-siRNA-2 (GA-2)	5′-CCACTTCGTCAAGCTCATT-3′
GAPDH-siRNA-3 (GA-3)	5′-CCTCAAGATCGTCAGCAAT-3′
MPC1-siRNA-1 (MP-1)	5′-CGGACTATGTCCGGAGCAA-3′
MPC1-siRNA-2 (MP-2)	5′-GGAGCAAGGACTTCCGCGA-3′
MPC1-siRNA-3 (MP-3)	5′-TTCCGCGACTACCTCATGA-3′
NDUFV1-siRNA-1 (ND-1)	5′-GTACAAGACAAAGGAGATT-3′
NDUFV1-siRNA-2 (ND-2)	5′-CCAAGTATCTGGTGGTGAA-3′
NDUFV1-siRNA-2 (ND-3)	5′-CTCGACGGACATCGTGAAA-3′
LDHB-siRNA-1 (LD-1)	5′-GAAAGTCTCTGACGGATGA-3′
LDHB-siRNA-2 (LD-2)	5′-CTGGAAGCTAAGTGGATTA-3′
LDHB-siRNA-3 (LD-3)	5′-GTGCCTACGAAGTCATCAA-3′

### Western blotting

Western blotting of CCs was performed as reported previously^47^. (i) A radioimmuno-precipitation assay (RIPA) buffer was prepared, which contained 150 mM NaCl, 1.0% Triton X-100, 0.5% sodium deoxycholate, 0.1% SDS, 1 mM phenylmethyl sulfonyl fluoride, and 50 mM Tris (pH 8). (ii) CCs from 2 wells of monolayer culture in a 96-well plate were washed in cooled PBS, and lysed in 20 µl RIPA buffer. (iii) After the total protein concentration was determined using a BCA Protein Assay Kit (P0012; Beyotime Institute of Biotechnology) and adjusted to 1 µg/µl, 20 µl were placed in a 0.5-ml microfuge tube and frozen at −80 °C until use. (iv) 5 µl of 5 × sodium dodecyl sulfate polyacrylamide gel electrophoresis (SDS-PAGE) loading buffer were added to each tube, and the tubes were heated to 100 °C for 5 min to extract protein. (v) To separate total proteins, a SDS-PAGE was run on a 10% polyacrylamide gel and the proteins obtained were transferred electrophoretically onto polyvinylidene fluoride membranes. (vi) The membranes were washed in TBST (150 mM NaCl, 2 mM KCl, 25 mM Tris and 0.05% Tween 20; pH 7.4), blocked for 2 h at 37 °C with TBST containing 3% BSA, and incubated at 4 °C overnight with primary antibodies. (vii) After being washed in TBST, the membranes were incubated for 1.5 h at 37 °C with secondary antibodies.

The primary antibodies used included rabbit anti-G6PD monoclonal antibodies (1:1000, ab993, Abcam Co., Ltd, Beijing, China), mouse anti-GAPDH monoclonal antibodies (1:1000, CW0100A, CWBio Co., Ltd, Beijing, China), rabbit anti-LDHB polyclonal antibodies (1:1000, 14824-1-AP, Proteintech Co., Ltd, Wuhan, China), rabbit anti-MPC1 polyclonal antibodies (1:200, TA315496S, ORIGENE Co., Ltd, Beijing, China), rabbit anti-NDUFV1 polyclonal antibodies (1:500, 11238-1-AP, Proteintech Co., Ltd, Wuhan, China), mouse anti-β-tubulin monoclonal antibodies (1:1000, 05–661, Merck Millipore), and mouse anti-β-actin monoclonal antibodies (1:1000, CW00096M, CWBio Co., Ltd). The secondary antibodies used included goat anti-rabbit IgG(1:500, CW0111, CWBio Co., Ltd, Beijing, China) and goat anti-mouse IgG (1:1000, CW0110, CWBio Co., Ltd). Signals were detected using a 5-bromo-4-chloro-3-indolyl phosphate/tetranitroblue tetrazolium chloride alkaline phosphatase color development kit (Beyotime Institute of Biotechnology). The sum density of each protein band image was analyzed using an Image-Pro Plus software (Media 225 Cybernetics, Inc., Silver Spring, MD, USA). The density value of each sample was divided by that of its internal control to get the sample protein/internal control ratio.

### Microinjection of DOs with siRNAs

Micromanipulation was performed using a Leica inverted microscope (Leica DM IRB, Leica Microsystems Wetzlar GmbH) equipped with differential interference contrast and two Leica mechanical micromanipulators. Pig oocytes at the germinal vesicle stage were freed of CCs as mentioned above and the resultant DOs were injected with MPC1, NDUFV1 or LDHB siRNAs in NCSU-23 supplemented with 10 mM Hepes and 15 mM pyruvate (for injection of MPC1 and NDUFV1 siRNAs) or 10 mM lactate (for injection of LDHB siRNAs). Briefly, 10 pl of 100 µM siRNAs were injected into each oocyte using a micropipette with an inner diameter of about 5 µm. Following injection, the DOs were cultured for 24 h in NCSU-23 containing 2 mM db-cAMP and 15 mM pyruvate or 10 mM lactate to prevent meiotic resumption and allow mRNA silencing. The oocytes were then cultured for 24 h in NCSU-23 containing 15 mM pyruvate or 10 mM lactate before examination for maturation.

### Enzyme-linked immunosorbent assay (ELISA) for ATP in medium conditioned by CCM

When CCs grew to about 80% of confluence, we transfected the monolayers with siRNAs for 48 h. We then replaced the spent medium in the wells with NCSU-23 containing 5.6 mM glucose or 15 mM pyruvate and cultured cells for 48 h. Then, we recovered the conditioned medium for ATP assay. We measured ATP contents using a porcine ATP Elisa kit (BLUE GENE, Shanghai, China). We added 100 µl of standards or samples to wells of a micro-titer plate pre-coated with porcine monoclonal antibodies, then added 50 µl of conjugate to each well and incubated them for 60 min at 37 °C. After we washed the micro-titer plate using the wash solution and dried using paper towels, we added 50 µl of substrate A and 50 µl of substrate B to each well and incubated for 15 min at 37 °C. We terminated the reaction by 50 µl of stop solution and measured the optical density within 15 min at 450 nm using a plate reader (BioTek-ELx808, BioTek Instruments, Inc.). We calculated the ATP concentrations in different media against the respective standard curves.

### Data analysis

Each treatment contained at least three replicates. Data were analyzed using ANOVA when each measure contained more than two groups or using t-test when each measure had only two groups. The differences were located by performing a Duncan multiple-comparison test during ANOVA. All the data were analyzed using the SPSS (Statistics Package for Social Sciences) software (SPSS 11.5, SPSS Inc. Chicago, IL), and were expressed as mean ± SEM. A difference was considered significant only when the p-value was less than 0.05.

## Supplementary information

Supplementary Information.
