# Supplementary material for: Effects of glucose metabolism pathways on nuclear and cytoplasmic maturation of pig oocytes
Source: Sci Rep. 2020 Feb 17;10:2782. doi: 10.1038/s41598-020-59709-6 (PMC7026050; doi:10.1038/s41598-020-59709-6)

Title: Effects of glucose metabolism pathways on nuclear and cytoplasmic maturation of pig oocytes

Running title: Glucose metabolism in pig oocytes

Jing Wen[2,3], Guo-Liang Wang[1,3], Hong-Jie Yuan[1], Jie Zhang[1], Hong-Li Xie[1], Shuai Gong[1], Xiao Han[1], and Jing-He Tan[1,2,4]

1. Shandong Provincial Key Laboratory of Animal Biotechnology and Disease Control and Prevention, College of Animal Science and Veterinary Medicine, Shandong Agricultural University, Tai'an City 271018, P. R. China

2. College of Life Science, Northeast Agricultural University, Harbin, 150030, P. R. China

3. These authors contributed equally to this work.

4. Corresponding author: Jing-He Tan, College of Animal Science and Veterinary Medicine, Shandong Agricultural University, Tai-an City 271018, Shandong Province, P R China; Phone: 0538-8249616; FAX: 0538-8241419; Email: tanjh@sdau.edu.cn

Original images of gels/blots from Western blotting

Fig. 3D

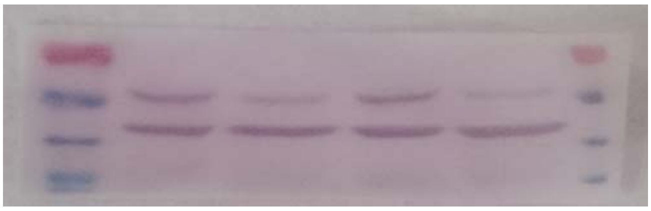

Fig. 3E

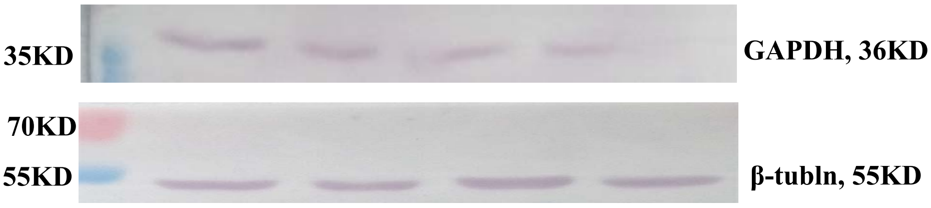

Fig. 4C

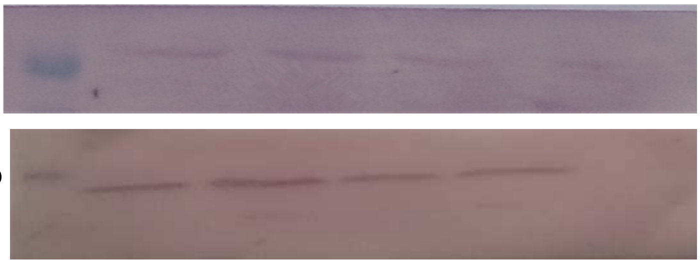

Fig. 4D

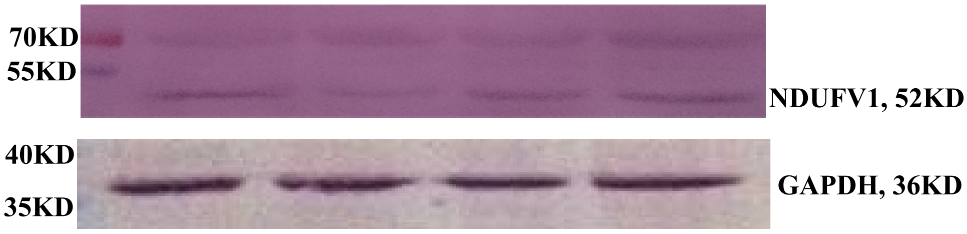

Fig. 4E

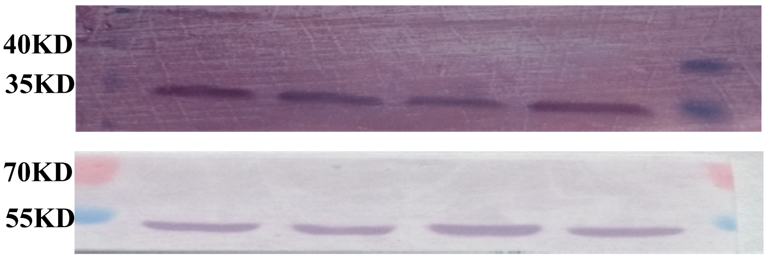

Supplement: Supplementary file 1 — Supplementary Information. [file 41598_2020_59709_MOESM1_ESM.pdf]
